# Effect of Essential Oils on Pathogenic Bacteria

**DOI:** 10.3390/ph6121451

**Published:** 2013-11-25

**Authors:** Filomena Nazzaro, Florinda Fratianni, Laura De Martino, Raffaele Coppola, Vincenzo De Feo

**Affiliations:** 1 Istituto di Scienze dell’Alimentazione, ISA-CNR, Via Roma 64, 83100 Avellino, Italy; E-Mails: fratianni@isa.cnr.it (F.F.); direttore@isa.cnr.it (R.C); 2 Dipartimento di Farmacia,Via Giovanni Paolo II, 132, 84084 Fisciano (SA), Italy; E-Mails: ldemarti@unisa.it (L.D.M.); defeo@unisa.it (V.D.F.)

**Keywords:** essential oils, antimicrobial, cell membrane, microbial morphology, metabolome, fatty acids, quorum sensing

## Abstract

The increasing resistance of microorganisms to conventional chemicals and drugs is a serious and evident worldwide problem that has prompted research into the identification of new biocides with broad activity. Plants and their derivatives, such as essential oils, are often used in folk medicine. In nature, essential oils play an important role in the protection of plants. Essential oils contain a wide variety of secondary metabolites that are capable of inhibiting or slowing the growth of bacteria, yeasts and moulds. Essential oils and their components have activity against a variety of targets, particularly the membrane and cytoplasm, and in some cases, they completely change the morphology of the cells. This brief review describes the activity of essential oils against pathogenic bacteria.

## 1. Introduction

The increasing resistance of microorganisms to conventional chemicals and drugs has prompted scientists to search for novel sources of biocides with broad-spectrum activities [1]. Since ancient times, plants and their derivatives, such as essential oils (EOs), have been used in folk medicine. In nature, EOs play an important role in the protection of plants. They also may attract some insects to promote the dispersion of pollens and seeds or keep away other undesirable insects. Thus, EOs can play a role in mediating the interactions of plants with the environment [2]. EOs are concentrated natural products with strong smells that are produced by aromatic plants as secondary metabolites. These oils are present as variable mixtures of primarily terpenoids, especially monoterpenes (C10) and sesquiterpenes (C15), although diterpenes (C20) may also be present. A variety of other molecules also occur, such as acids, alcohols, aldehydes, aliphatic hydrocarbons, acyclic esters or lactones; rare nitrogen- and sulphur-containing compounds; coumarins; and homologues of phenylpropanoids. EOs are liquid, volatile, limpid and coloured and are soluble in lipids and organic solvents that have a lower density than water. They can be present in all plant organs, including buds, flowers, leave, seeds, twigs, stems, flowers, fruits, roots, wood or bark, but are generally stored by the plant in secretory cells, cavities, canals, glandular trichomes or epidermic cells. EOs are extracted from various aromatic plants that are generally found in temperate or warm countries, where they often represent an important part of the traditional pharmacopoeia. These plants may be known for their antioxidant effects as well as their antiseptic and medicinal properties and fragrance and are often used in the preservation of foods and as analgesics, sedatives, anti-inflammatories, spasmolytics and local anaesthetics [2]. EOs contain a wide series of secondary metabolites that can inhibit or slow the growth of bacteria, yeasts and moulds [3,4,5]. The EOs and their components have a variety of targets, particularly the membrane and cytoplasm, and in certain situations, they completely alter the morphology of the cells. This brief review will describe the activity of EOs against pathogenic bacteria.

## 2. Activity of Essential Oils against Bacteria

**Figure 1:**
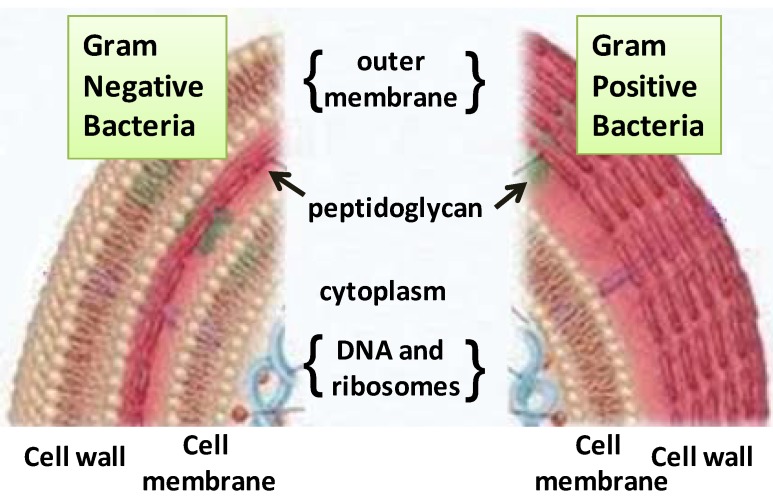
Schematic of the envelopes of Gram-positive (on the right) and Gram-negative bacteria (on the left).

Generally, Gram-negative bacteria are more resistant to EOs than Gram-positive bacteria [6]. Before examining the effects of EOs on bacteria, we should briefly consider the differing structures of the cell walls of Gram-positive and Gram-negative bacteria. Approximately 90%–95% of the cell wall of Gram-positive bacteria consists of peptidoglycan, to which other molecules, such as teicoic acid and proteins, are linked (Figure 1).

The structure of the Gram-positive bacteria cell wall allows hydrophobic molecules to easily penetrate the cells and act on both the cell wall and within the cytoplasm. Phenolic compounds, which are also present in the EOs, generally show antimicrobial activity against Gram-positive bacteria. Their effect depends on the amount of the compound present; at low concentrations, they can interfere with enzymes involved in the production of energy, and at higher concentrations, they can denature proteins [7]. The cell wall of Gram-negative bacteria is more complex. It has a peptidoglycan layer that is 2–3 nm thick, which is thinner than in the cell wall of Gram-positive bacteria, and composes approximately 20% of the dry weight of the cell. An outer membrane (OM) lies outside of the thin peptidoglycan layer. The peptidoglycan and OM are firmly linked by Braun’s lipoprotein; this protein is covalently bound to the peptidoglycan and is embedded in the OM. The presence of an OM is one of the features that differentiate Gram-negative from Gram-positive bacteria. It is composed of a double layer of phospholipids that is linked to inner membrane by lipopolysaccharides (LPS). The peptidoglycan layer is covered by an OM that contains various proteins as well as LPS. LPS consists of lipid A, the core polysaccharide, and the O-side chain, which provides the “quid” that allows Gram-negative bacteria to be more resistant to EOs and other natural extracts with antimicrobial activity. Small hydrophilic solutes are able to pass through the OM via abundant porin proteins that serve as hydrophilic transmembrane channels, and this is one reason that Gram-negative bacteria are relatively resistant to hydrophobic antibiotics and toxic drugs [8,9]. The OM is, however, almost but not totally impermeable to hydrophobic molecules, some of which can slowly traverse through porins [10,11]. The mechanisms of action of EOs and/or their components are dependent on their chemical composition. For instance, thymol and carvacrol have similar antimicrobial effects but have different mechanisms of action against Gram-positive and Gram-negative bacteria. The location of one or more functional groups on these molecules can affect their antimicrobial activity. Thymol is structurally analogous to carvacrol, but the locations of the hydroxyl groups differ between the two molecules. However, these differences do not affect the activity of either antimicrobial agent. The antimicrobial activity of other molecules, such as limonene and *p*-cymene, depends on the alkyl group. Thus, in some cases, limonene can be considered to be more effective than *p*-cymene [12]. EOs and/or their constituents can have a single target or multiple targets of their activity. For instance, *trans*-cinnamaldehyde can inhibit the growth of *E. coli* and *S. typhimirium* without disintegrating the OM or depleting intracellular ATP. Similar to thymol and carvacrol, *trans*-cinnamaldehyde likely gains access to the periplasm and deeper portions of the cell. Carvone is also ineffective against the OM and does not affect the cellular ATP pool [13,14]. It is difficult to predict the susceptibility not only of a certain species but also a certain strain within the same species to the EOs. De Martino *et al.* [5,15] observed that two strains of *Bacillus cereus* behaved differently when exposed to the same EOs and their singular components. Identifying the mode of action of EOs requires much study of the raw material until the singular components are identified, and the mode of action should also be studied in multiple strains and species of microorganisms. Expanding our basic knowledge of the molecules present in the EOs will support future studies into the comprehensive modes of antimicrobial action of EOs.

### 2.1. Terpenes

Terpenes are hydrocarbons that are formed through the combination of several isoprene units (C5H8). They are synthesised within the cytoplasm of the vegetal cell; their synthesis occurs in the mevalonic acid pathway starting from acetyl CoA. Terpenes contain a hydrocarbon backbone that can be rearranged into a cyclic structure by cyclases [16]. The most common terpenes are monoterpenes (C_10_H_16_) and sesquiterpenes (C_15_H_24_), but longer chains, such as diterpenes (C_20_H_32_), triterpenes (C_30_H_40_) and so on, are also present in the plant cell. Among the terpenes, p-Cymene, limonene, terpinene, sabinene and pinene are the most well known. Most terpenes do not possess high inherent antimicrobial activity. *p*-Cymene, one of the most important components of thyme EO, does not show antimicrobial activity against many Gram-negative pathogens [17]. Other terpenes, such as limonene, α-pinene, β-pinene, γ-terpinene δ-3-carene, (+)-sabinene and α-terpinene showed a very low or no antimicrobial activity against 25 genera of bacteria [12]. These *in vitro* tests indicate that terpenes show ineffective antimicrobial activity when used as singular compounds.

### 2.2. Terpenoids

Terpenoids are terpenes with added oxygen molecules or that have had their methyl groups moved or removed by specific enzymes [16]. Thymol, carvacrol, linalool, menthol, geraniol, linalyl acetate, citronellal and piperitone are the most common and well-known terpenoids. The antimicrobial activity of most terpenoids is related to their functional groups, and the hydroxyl group of the phenolic terpenoids and the presence of delocalised electrons are important elements for their antimicrobial action. For example, carvacrol is more effective than other EOs, such as *p*-cymene [12,18,19]. The exchange between the hydroxyl group and a methyl ether in carvacrol can affect its hydrophobicity and antimicrobial activity. The position of the hydroxyl group in the phenolic molecule does not affect the trend of the antimicrobial action. Compared with carvacrol, thymol has similar antimicrobial activity against *B. cereus*, *S. aureus* and *P. aeruginosa*, even though its hydroxyl group is located in a different position [18,20]. Thymol and carvacrol have prominent OM disintegrating properties. Helander *et al.* [13] demonstrated that enhanced LPS release and sensitised cells to detergents. However, thymol and carvacrol do not directly act as OM permeabilising agents (unlike EDTA or polyethylenimine, which disintegrate the OM at sub-lethal concentrations) [9,14]. These compounds are also capable of increasing the permeability of the cytoplasmic membrane to ATP. *p*-Cymene is the precursor of carvacrol and is a monoterpene with a benzene ring without any functional groups on its side chains. Others have described the antimicrobial activity of p-cymene when it is used alone [17,21,22], and *p*-cymene can also increase the antimicrobial activity of other compounds, such as its derivative carvacrol [18,23]. *p*-Cymene shows a high affinity for microbial membranes and can perturb the membranes, causing them to expand and affecting the membrane potential of intact cells [18]. *p*-Cymene does not affect the membrane permeability but may decrease the enthalpy and melting temperature of membrane [24]; these properties strengthen the notion that this compound may act as a substitutional impurity in the membrane. However, *p*-cymene does not act solely at the membrane level. Burt *et al.* [25] demonstrated that although the compound did not affect protein synthesis in *E. coli*, it did affect the membrane potential. Treatment with p-cymene resulted in decreased cellular motility because the proton motive force is required for flagellar movement. Thymol is a phenolic monoterpenoid that is found in the EO of thyme. Its structure is similar to carvacrol, and it has hydroxyl groups occupying different positions on the phenolic ring. Similar to carvacrol, thymol antimicrobial activity results in structural and functional alterations in the cytoplasmic membrane [26] that can damage the outer and inner membranes; it can also interact with membrane proteins and intracellular targets. The interaction of thymol with the membrane affects membrane permeability and results in the release of K+ ions and ATP [20,27,28]. In some cases, thymol can induce the release of lipopolysaccharides, but it does not affect chelating cations [13]. Thymol integrates within the polar head-groups of the lipid bilayer, inducing alterations of the cell membrane. At low levels of thymol, the membrane can adapt its lipid profile to maintain membrane function and structure [29]. Thymol also interacts with proteins, as demonstrated using a model system with bovine serum albumin [30]. The interactions of thymol with proteins occur at different sites within the cell and can affect a variety of cellular functions. Carvacrol is a phenolic monoterpenoid that is found primarily in the EO of oregano. Along with compounds such as thymol, carvacrol is one of the most investigated EO constituents. Similar to thymol, carvacrol acts on microbial cells and causes structural and functional damage to their membranes [26] that results in increased permeability. Carvacrol is one of the few components of an EO that has a disintegrating effect on the OM of Gram-negative bacteria [31]. It causes the release of LPS [13] and also acts on cytoplasmic membrane to alter the transport of ions. The activity of carvacrol seems to be linked to the presence of a hydroxyl group that may function as a trans-membrane carrier of monovalent cations by carrying H+ into the cell cytoplasm and transporting K+ back out [18,19]. This hypothesis conflicts with other reports that the antimicrobial activity of carvacrol is not linked to the hydroxyl groups but is instead related to the presence of non-hydroxyl groups [32]. However, the mode of action of carvacrol seems to be to increase the fluidity and permeability of membranes. When microbial cells are exposed to carvacrol, they may change their membrane fatty acid composition. This is a well-known mechanism that allows cells to maintain optimal membrane structure and function. The alteration of the composition of fatty acids in response to carvacrol could affect not only membrane fluidity but may also subsequently affect its permeability [8,33,34,35]. Carvacrol’s effect on membrane permeability was confirmed monitoring the efflux of H+, K+, carboxyfluorescein and ATP and the influx of nucleic acid stains [13,20,24,28,36]. There is also limited evidence that carvacrol affects periplasmic enzymes and membrane proteins [30], and it may also have intracellular targets [37]. Carvacrol can affect the folding or insertion of OM proteins. Burt *et al.* [25] showed that *E. coli* cells grown in the presence of a sub-lethal concentration of carvacrol produced significantly more GroEL, indicating that carvacrol affected protein folding. Carvacrol also inhibited the synthesis of another microbial protein, flagellin, and gave rise to cells without flagella that subsequently exhibited decreased motility. However, even cells with flagella exhibited decreased motility that was depended upon the amount of carvacrol, indicating that the compound also diminished the proton motive force needed to drive flagellar movement [38].

### 2.3. Phenylpropenes

Phenylpropenes are named as such because they contain a six-carbon aromatic phenol group and a three-carbon propene tail from cinnamic acid, which is produced during the first step of phenylpropanoid biosynthesis. These compounds represent a relatively small portion of EOs. Eugenol, isoeugenol, vanillin, safrole and cinnamaldehyde are the most studied phenylpropenes. Most antimicrobial activity of these molecules is conferred by their free hydroxyl groups [39]. The antimicrobial activity of eugenol can be ascribed to the presence of a double bond in the α,β positions of the side chain and to a methyl group located in the γ position [40]. The antimicrobial activity of the phenylpropenes also depends on the type and number of substitutions on the aromatic ring and similar to most other EOs, on the microbial strain and conditions in which the EO is tested [41]. Generally speaking, the phenylpropenes show a range of antibacterial activity. Isoeugenol is more active against bacteria than eugenol and is also effective against yeasts and moulds [39,42,43]. Interestingly, eugenol and isoeugenol exhibit higher activity against Gram-negative bacteria than Gram-positive bacteria [44]. Cinnamaldehyde is generally less powerful than eugenol [45], but when it is used against *E. coli* and *S. typhimurium*, its activity is similar to thymol and carvacrol, the most potent EOs [13]. Eugenol alters the membrane, affects the transport of ions and ATP and changes the fatty acid profile of different bacteria. It also acts against different bacterial enzymes, including ATPase, histidine carboxylase, amylase and protease [46,47]. Cinnamaldehyde has at least three mechanisms of action against bacteria. At low concentrations, it inhibits enzymes involved in cytokine interactions or other less important cell functions, and at higher concentrations, it acts as an ATPase inhibitor. At a lethal concentration, cinnamaldheyde perturbs the membrane. Some studies have reported conflicting information on the membrane-perturbing activity of cinnamaldheyde. For example, a sub-lethal concentration of the molecule does not affect the integrity of the membrane in *E. coli* but can inhibit the growth and bioluminescence of the microorganism *Photobacterium leiognathi*; this suggests that cinnamaldheyde gains access to the periplasm and perhaps also to the cytoplasm [13]. Cinnamaldehyde is indeed capable of altering the lipid profile of the microbial cell membrane [47]. Vanillin is a phenylpropene phenolic aldehyde with a poorly understood mechanism of action. However, some studies have shown that it inhibits the respiration pathway in *E. coli* and *L. innocua* and that it has activity against some lactic acid bacteria, such as *L. plantarum*, by disrupting K+ and pH homeostasis [48]. Fitzgerald *et al.* [49] observed that vanillin’s primary target is the membrane but reported that other targets may be present within the microbial cell. Carvone is capable of disrupting the pH gradient and membrane potential of cells. With increasing amount of carvone, Oosterhaven *et al.* [50] observed a decrease in the growth rate of *E. coli*, *Streptococcus thermophilus* and *L. lactis* and hypothesised that the compound might act by disturbing the metabolic energy status of cells. In contrast, another study [13] found that carvone was ineffective against the OM of *E. coli* and *S. typhimurium* and did not affect their intracellular ATP pool. γ-Terpinene does not affect the growth of S. typhimurium [30], whereas α-terpinene inhibited 11 of the 25 bacterial species screened by Dorman and Deans [12].

## 3. Mechanisms of Action of the Essential Oils and/or Their Components

The antimicrobial activity of EOs, similar to all natural extracts, is dependent on their chemical composition and the amount of the single components. Many of the antimicrobial compounds are constitutively expressed by the plants, and others can be synthesised as mechanism of self-defence in response to pathogens. Vegetables, spices and fruits with high level of EOs are excellent sources of natural elements with activity against microorganisms of agricultural and health interest [51]. These molecules can be naturally present in their active form in the plant or can be activated by specific enzymes when the vegetal organism is subjected to particular biotic or abiotic stress [52]. Different amounts of specific compounds can affect the antimicrobial activity of EOs. For example, high concentrations of cinnamic aldehyde, eugenol or citral confer antimicrobial properties to EOs [53,54]. The monoterpenes and phenols present in thyme, sage and rosemary EOs possess noticeable antimicrobial, antifungal and antiviral activity [55,56,57]. Some EOs, such as those found in basil, sage, hyssop, rosemary, oregano and marjoram, are active against *E. coli*, *S. aureus*, *B. cereus* and *Salmonella* spp. but are less effective against *Pseudomonas* spp. due to the formation of exopolysaccharides that increase resistance to EOs [5,57]. The mechanism of action of EOs depends on their chemical composition, and their antimicrobial activity is not attributable to a unique mechanism but is instead a cascade of reactions involving the entire bacterial cell [57]; together, these properties are referred to as the “essential oils versatility”. In general, EOs act to inhibit the growth of bacterial cells and also inhibit the production of toxic bacterial metabolites. Most EOs have a more powerful effect on Gram-positive bacteria than Gram-negative species, and this effect is most likely due to differences in the cell membrane compositions (Figure 1) [3,56,58].

## 4. What are the Possible Mechanisms of Action of the EOs and/or Their Components against Microbes?

**Figure 2:**
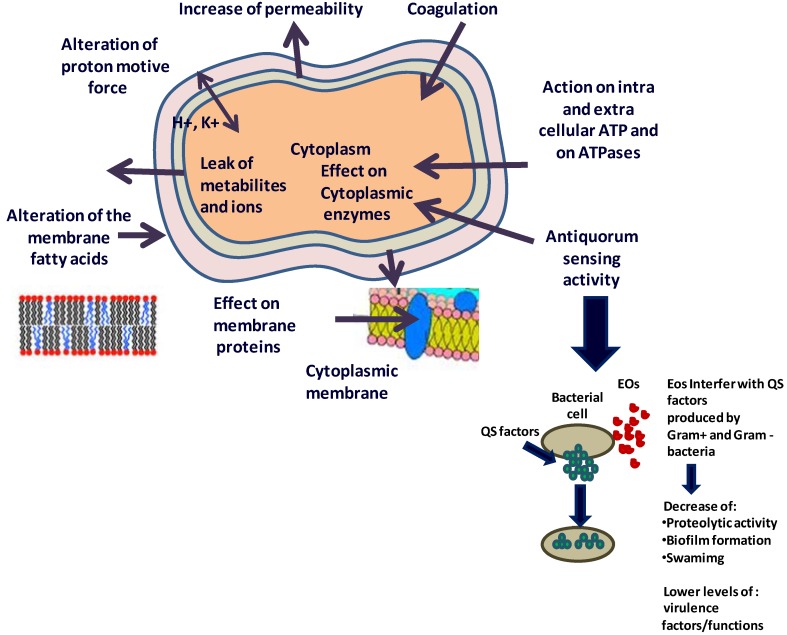
Mechanism of action and target sites of the essential oils on microbial cells.

Diverse mechanisms have been described to explain the activity of an EO on bacterial cells. The activity of an EO can affect both the external envelope of the cell and the cytoplasm. The hydrophobicity that is typical of EOs is responsible for the disruption of bacterial structures that leads to increased permeability due to an inability to separate the EOs from the bacterial cell membrane. The permeability barrier provided by cell membranes is indispensable to many cellular functions, including maintaining the energy status of the cell, membrane-coupled energy-transducing processes, solute transport and metabolic regulation. The cell membrane is also essential for controlling the turgor pressure [59,60]. Toxic effects on membrane structure and function are generally used to explain the antimicrobial activity of EOs [61,62,63]. In fact, the mechanisms of action of the EOs include the degradation of the cell wall [13,64], damaging the cytoplasmic membrane, cytoplasm coagulation [18,33,65], damaging the membrane proteins, increased permeability leading to leakage of the cell contents [20,30], reducing the proton motive force [66], reducing the intracellular ATP pool via decreased ATP synthesis and augmented hydrolysis that is separate from the increased membrane permeability and reducing the membrane potential via increased membrane permeability [57]. Helander *et al.* [13] described the effects of different EO components on the OM permeability. Tea tree oil induced damage to the cell membrane structures that was accompanied by decreased viability for all three microorganisms included in their study, and the membrane damage was confirmed as the most likely cause of cell death. Thus, the hydrophobic nature of EOs allows them to penetrate microbial cells and cause alterations in its structure and functionality. This could explain why EOs are generally most effective, with some exceptions [67], against Gram-positive microorganisms. The external capsule of some Gram-negative bacteria limits or prevents the penetration of EOs into the microbial cell. The compounds present in the EOs are also capable of interfering with proteins in the wall that are often involved in the transport of essential molecules into the cell. Other authors have proposed that the components of the EO act in different manners to result in the loss of microbial viability. The effects of EOs usually lead to the destabilisation of the phospholipid bilayer, the destruction of the plasma membrane function and composition, the loss of vital intracellular components and the inactivation of enzymatic mechanisms. In some cases, essential oils also alter membrane permeability by destroying the electron transport system [68], and a number of components of the EOs, such as carvon, thymol and carvacrol, lead to an increase in the intracellular concentration of ATP, an event that is linked to the destruction of the microbial membrane [13]. Inhibiting electron transport for energy production and disrupting the proton motive force, protein translocation and synthesis of cellular components are all physiological changes that can result in cell lysis and death [19,29]. The integrity of the cell membrane is essential for the survival of bacteria because it is a key element for the fundamental biological activities taking place within the cells. The membrane represents an effective barrier between the cytoplasm and the external environment; the import and export of the metabolites and ions essential for all activities occurring in the microbial cell occur through the cell membrane. When antimicrobial compounds are present in the environment surrounding microorganisms, the bacteria may react by altering the synthesis of fatty acids and membrane proteins to modify the fluidity of the membrane [69]. The hydrophobicity of the EOs and their components allow them to diffuse through the double lipid layer of the membrane. The EOs can alter both the permeability and function of membrane proteins. Some EOs, particularly oils that are rich in phenolics, are able to insert into the phospholipids bilayer of bacterial cell walls, where they bind to proteins and prevent them from performing their normal functions [30]. This phenomenon indicates that the membrane is the first target of EOs. As previously reported, the mechanism of action of the EO is not isolated but instead involves a series of events both on the cell surface and within the cytoplasm. The alteration of membrane permeability and the defects in the transport of molecules and ions result in a “disbalance” within the microbial cell. This subsequently leads to cytoplasm coagulation, the denaturation of several enzymes and of cellular proteins and the loss of metabolites and ions [4]. In many conditions, such as in the presence of sub-lethal concentrations of EOs or other antimicrobial compounds, microorganisms react by increasing their expression of the stress-response proteins [25] to repair the damaged proteins [20]. However, when the concentration of EOs or other natural antimicrobials is higher, this response is unable to prevent cell death. This effect is more evident for Gram-positive bacteria. The cell wall of Gram-negative bacteria is more resistant to the activity of EOs and their components. The Gram-negative cell wall does not allow for the entrance of hydrophobic molecules as readily as Gram-positive bacteria; thus, EOs are less able to affect the cell growth of the Gram-negative bacteria [4]. Because of the wide variety of molecules present in the natural extracts, the antimicrobial activity of the EOs cannot be attributed to a single mechanism. Instead, different biochemical and structural mechanisms are involved [70] at multiple sites within the cell and on the cell surface. These mechanisms include chemical modifications of the cell membrane, cytoplasm, enzymes and proteins, and they can completely change the conformation of the microbial cell. Furthermore, the sustained loss of ions or metabolites due to exposure to an EO can compromise the microbial metabolism and lead to cell death [4,57]. For example, tea tree oil can cause *E. coli* to die without lysing the cell [65]. Figure 2 describes some potential mechanisms of action of the EOs and/or their components and shows the potential cell targets of their antimicrobial activity. However, each of these actions cannot be considered separate events but instead may be a consequence of the other activities.

### 4.1. Effect on the Fatty Acids Profile of the Cell Membrane

The lipid biosynthesis pathway is an important target for the development of novel antimicrobials [71,72]. Due to their hydrophobic nature, EOs and/or their components can affect the percentage of unsaturated fatty acids (UFAs) and alter their structure [4]. The adaptation of the cells to the presence of these compounds at a concentration lower than the MIC could result in an increase in the percentage of UFAs responsible for the fluidity of the membrane [35,71], which may indicate a certain mechanism of action against the outer cell envelope that could cause membrane structural alterations observable by SEM. The lipid profile of resting cells shows reorganisation after treatment with EO compounds, and many authors have described the bacterial fatty acid synthesis pathway as an optimal target of many antibacterial agents [73,74,75]. Treatment with thymol, carvacrol and eugenol, which are all phenolic compounds, may increase the amount of saturated C16 (and shorter length) fatty acids, increase the amount of saturated C18 and decrease the amount of unsaturated C18 fatty acids in the bacterial membrane. When saturated C18 fatty acids are absent, a decrease in C18:2 *trans* and C18:3 *cis* may occur after the treatment (e.g., *S. typhimurium*), or an increase of C18:1 *cis* and a corresponding decrease of C18:2 *trans* may be observed (e.g., *B. thermosphacta*). In addition to direct effects on the fatty acids of the OM, EOs may also affect the enzymes that are involved in fatty acid synthesis, such as a multicomponent membrane desaturase enzyme that is generally employed by cells to produce saturated fatty acids (SFAs) [35,76,77]. Again, the activity of the EOs and/or their components is not attributable to a single event; most of the components of the EOs [13,18,48] act on the OM and increase its permeability. This results in the dispersion of the desaturase enzymes and allows them to act on the membrane fatty acids. Conversely, EOs do not activate other enzymatic systems, such as the *cis-trans* isomerase that regulates the conversion of *cis* fatty acids into their *trans* isomers. This enzyme is generally active during the adaptation of cells to environmental stresses [78]. On the whole, the activity of EO components is most likely do to simultaneous effects on a pool of fatty acid enzymes that lead to an increase in the cis isomers, a reduction of the chain length and a general decrease in the abundance of UFAs. An increase in the amount of SFAs in the membrane lipid bilayer results in a loss of membrane fluidity and a consequent increase in membrane rigidity [74]. In some cases, cells defective in the production of UFAs can continue to grow and synthesise phospholipids, but they eventually begin to lose metabolites and subsequently lyse. In some microbial cells, such as *B. thermosphacta* and *S. typhimurium*, a reduction in the levels of *trans* C18 fatty acid is not accompanied by an increase in its *cis* isomer, but the increase of the C18:1 *cis* and C17:1 *cis* fatty acids may be related to such a decrease [35].

### 4.2. Action on Proteins

Different components of EOs can act on proteins present in bacteria and may affect cell division. Cinnamaldehyde, for example, is capable of inhibiting cell separation in *B. cereus*. Bacterial cell division is regulated by FtsZ, a prokaryotic homolog of tubulin. FtsZ assembles into a Z-ring at the site of cell division; cinnamaldehyde can decrease the *in vitro* assembly reaction and bundling of FtsZ. It also can perturb the Z-ring morphology *in vivo* and reduce the frequency of the Z-ring per unit of cell length of *E. coli*. In addition, GTP-dependent FtsZ polymerisation is inhibited by cinnamaldehyde [79]. Cinnamaldehyde can also inhibit the rate of GTP hydrolysis and binds to FtsZ with favourable enthalpic interactions. After mapping the cinnamaldehyde binding region of FtsZ by saturation transfer difference–nuclear magnetic resonance and an *in silico* docking model, Domadia *et al.* [79] predicted the cinnamaldehyde binding pocket at the C terminal region involving the T7 loop of FtsZ and hypothesised that cinnamaldehyde binds to FtsZ, perturbs the cytokinetic Z-ring formation and inhibits its assembly dynamics. *S. enterica* serovar Thompson MCV1 grown in the presence of a sub-lethal concentration of thymol showed a different proteomic profile compared with the control [80]. The MALDI-TOF analysis showed that many proteins can be either up-regulated or down-regulated by the presence of thymol, with significant changes in proteins belonging to different functional classes. For example, the thioredoxin-1 (which belongs to a class of small E12 kDa redox active proteins and is essential for the regeneration of methionine sulfoxide reductase) was not expressed in the presence of thymol, indicating that its absence was due to the presence of the EO. Kumar *et al.* [81] hypothesised that Trx1 plays a role *E. coli* cell division because of its subcellular localisation. Different chaperon proteins can be concurrently up-regulated or newly synthesised. GroEL and DnaK are two examples of key proteins involved in protecting cells from thermal stress and promoting protein folding by binding to short, extended peptides in an ATP-dependent cycle. Thymol increases the expression of the chaperone proteins. This process begins in the lag phase, but chaperones keep working in the subsequent phases of the bacterial growth to help *Salmonella* adapt to adverse environmental conditions [82]. Treatment with thymol causes the up-regulation of some proteins and can trigger bacterial envelope stress due to the accumulation of mis-folded OM proteins. Another protein influenced by the presence of EOs, such as thymol, is the protein involved in the phosphotransferase system that is decreased by more than a half of its usual concentration. Thymol up-regulates the OM channel protein TolC that is involved in the formation of an efflux system that represents [83] an important mechanism of resistance to detergents and bile salts in *Salmonella* and may also be involved in resistance to thymol. Thymol can also affect the expression of proteins involved in energy metabolism, such as enolase, which is more than 10-fold up-regulated in thymol-treated cells, the 2,3-bisphosphoglycerate-dependent phosphoglycerate mutase dPGM and the glyceraldehyde-3-phosphate dehydrogenase A, which are down-regulated [82]. Some enzymes, such as those involved in glutamine transport, are overexpressed by *S. typhimurium* in the presence of thymol in an attempt to increase the virulence of the bacteria [84]. Other proteins, such as the DNA-binding protein H-NS and the 50S ribosomal proteins L7/L12, are down-regulated by thymol. This increases bacterial DNA stability and inhibits the transcription as further mechanism of protection. Thymol may also impair the citrate and acetate pathways and can influence some of the enzymes involved in the catabolism of different carbon and nitrogen sources. Proteins that are required for bacterial survival and are conserved among pathogens are considered excellent targets for inhibitor screening [85,86]. One such *E. coli* protein, YidC, a 60-kDa membrane protein [87], is essential for membrane protein translocation and insertion and acts with the Sec-translocase and separately to facilitate the insertion of proteins into the cell membrane. YidC represents an evolutionarily conserved protein that is required for the growth of *E. coli* [88,89]. YidC depletion leads to a decrease in the functional assembly of cytochrome o oxidase and F1Fo ATPase and a strong reduction in the proton motive force in *E. coli* [90]. Because it is a common feature of both Gram-negative and Gram-positive bacteria, YidC is an attractive target for the development of broad-spectrum antibacterial agents. Patil *et al.* [91] explored the feasibility and efficacy of using antisense-mediated gene silencing to specifically down-regulate YidC in *E. coli* and found that decreasing YidC expression made the microorganism more sensitive to the activity of carvacrol and eugenol. Their results showed that the YidC antisense-expressing clone was sensitised to the membrane disintegrating and membrane-bound ATPase-inhibiting antibacterial EOs eugenol and carvacrol; thus, the essential gene YidC may represent a therapeutic target of the antibacterial EOs eugenol and carvacrol.

### 4.3. Effect on ATP and ATPases

The production of ATP in prokaryotes occurs in both the cell wall and cytosol by glycolysis, and a correlation between the intracellular and extracellular ATP concentration has been demonstrated. EOs disrupt the cell membrane alter the intracellular and external ATP balance such that ATP is lost through the disturbed membrane [12,92,93]. The treatment of *E. coli* 0157:H7 strain EDL 933 and *Salmonella enterica* subsp *enterica* serovar *typhi* strain ATCC 19430 with mustard EO resulted in the loss of intracellular ATP in *S. aureus* [94]. The use of oregano EO in combination with the irradiation of *L. monocytogenes* and *S. aureus* resulted in a more significant reduction of their intracellular ATP levels [95,96]. Other intracellular events may contribute to the intracellular ATP decrease; for example, inorganic phosphate may have been lost by passing through the compromised permeable membrane [93,94,95,96,97], or the proton motive force and changes in the balance of some essential ions, such as K+ and H+, may have been disrupted [36]. The treatment of some pathogens, such as *E. coli* and *L. monocytogenes*, with eugenol, cinnamaldehyde and carvacrol inhibited the generation of adenosine triphosphate from dextrose and disrupted the cell membrane. An analysis of the intracellular and extracellular ATP levels of cells treated with eugenol, cinnamaldehyde and carvacrol suggested that these compounds might inhibit the ATPase activity of bacterial cells. However, although similar concentrations of eugenol or carvacrol (5 to 10 mM) may have a bactericidal effect on *E. coli* and *L. monocytogenes*, there are large differences in response between the three organisms to cinnamaldehyde, which has bactericidal activity against *E. coli* and *L. monocytogenes,* at 10 mM and 30 mM concentrations [45]. A possible explanation for the difference in the behaviour of the three bacterial species in response to cinnamaldehyde is that there may be differences in the ability of this small hydrophobic molecule to interact with the outer surface of the cells and thus gain access to the cell membrane. Some components of the EOs, such as eugenol, carvacrol and cinnamaldehyde, are capable of inhibiting the membrane-bound ATPase activity of *E. coli* and *L*. *monocytogenes*. Bacterial membranes have multiple enzymes with ATPase activity, including ATP-dependent transport proteins and the F1F0 ATPase that is involved in ATP generation and cellular pH regulation [26]. Gill and Holley [64] hypothesised that ATPase inhibition could represent a secondary factor rather than a primary cause of cell death. However, they also suggested that ATPase inhibition may play a significant role in reducing the growth rate at sub-lethal concentrations. Non-specific inhibition of membrane-bound or -embedded enzymes can be caused by small hydrophobic molecules as a result of changes in the protein conformation. This mechanism may cause the inhibition of ATPase activity, as well as the inhibition of other enzymes and altered bacterial growth [13,27,47,96,98].

### 4.4. Effect on the Metabolome

Intracellular and extracellular metabolomics have some fundamental advantages in that they can provide important information about functional genomics, metabolic engineering, strain characterisation, and cell communication mechanism. Microbial metabolites can change in response to environmental conditions [99,100]. Recently, several powerful standard analytical approaches, including as NMR, microarray, GC–MS, LC-MS, have been used to analyse the metabolome of bacteria subjected to environmental stress [101,102,103,104,105,106]. These techniques are increasingly used to investigate the metabolic effects of natural molecules with bacteriostatic and/or bactericidal activity. Most of the molecules that have been studied and/or identified by these techniques are major components of cell extracts and are easily identifiable because their signals in the NMR spectra do not overlap with others. Each individual metabolite responds differently to varying doses of the EOs or their components. For example, some effects might occur at low amounts of carvacrol, others only at higher doses. Picone *et al.* [107] observed that most of the signals obtained by NMR changed in intensity in response to increasing amounts of carvacrol in *E. coli*. They found that glucose tends to accumulate when microbial cells are treated with carvacrol, and the inability of the cells to metabolise the glucose leads to a loss of viability. In contrast, organic acids, with the exception of formate, showed a significant decrease in concentration that was inversely proportional to the dose of carvacrol. Formate increased until a certain dose of carvacrol and then drastically decreased to nearly zero; the increase in formate (one of the main sugar molecules produced by *E. coli* during fermentation) may indicate a possible metabolic shift toward fermentation. At the highest carvacrol concentration, the irreversible loss of cell viability arrested the cell metabolism, resulting in the subsequent disappearance of formate. The concentration of aromatic amino acids remained stable at the lowest level of carvacrol but increased at higher doses. Other amino acids, such as alanine, were stable even at the highest amount of the EO. Overall, the metabolome analysis showed that the cellular processes affected by exposure to carvacrol were variable. When *E. coli* cells were treated with carvacrol, the glycolytic pathway modified the amounts of formate and succinate, two organic acids that are normally present in the Krebs cycle, which together with citrate, indicates a shift from respiration to fermentation and K+ leakage [108]. The exposure of *E. coli* BL21 cells to cinnamaldehyde resulted in the production of an increased number of metabolites, such as indole, alkanes, alcohol, acids, esters, dimethyl-disulphide and so on, during the mid-logarithmic growth phase [109] that could lead to tremendous cell stress, depending on level of exposure to cinnamaldehyde and on the cell density.

### 4.5. Effects on Cell Morphology

The activity of EOs and/or their components differs depending on the shape of the bacteria studied, and rod shaped bacterial cells have been reported to be more sensitive to EOs than coccoid cells. Generally, *S. typhimurium* and *E. coli* have a normal rod shape with a smooth surface, whereas *M. luteus* and *S. aureus* have a normal coccoid shape. After 24 h of treatment with the EO of mint, cellular damage of rod bacteria was observed by Hafedh *et al.* [110], whereas the damage was less evident in coccoid bacteria. These results are in agreement results of Kalchayanad et al [111], who showed morphological changes of two pathogens (*E. coli* 0157:H7 and *S. typhimurium*) when exposed to hydrostatic pressure stress and bacteriocins, and with the results of experiments performed by Braga and Ricci [112] on *E. coli* cells treated with cefodizime. These results led to the hypothesis that the exopolysaccharide on the OM of cells became detached and released or that the peptidoglycan and cytoplasmic membrane were perturbed [113]. Sikkema *et al.* [26,114] showed that as a result of their lipophilic character, cyclic monoterpenes are preferentially reparted from an aqueous phase into membrane structures. This results in membrane expansion, increased membrane fluidity and the inhibition of a membrane-embedded enzyme. Electron microscopy of *E. coli* cells after exposure to tea tree oil revealed a loss of electron-dense cellular material and showed coagulation of the cytoplasmic contents [111]; however, these effects were secondary events that occurred after cell death [65]. Kwon *et al.* [98] tested the effect of cinnamaldehyde on the morphology of *B. cereus* and found that without treatment, the bacteria appeared as well-separated rods. However, bacterial cells treated with cinnamaldehyde appeared as elongated, filamentous structures in which the cells did not appear to be separated from one another. The septa were present between the filamentous cells, but its formation was incomplete. Cells treated with cinnamaldehyde for 1 h showed a strong inhibition of cell separation and appeared as filamentous cells. The SEM analysis of different bacteria treated with eugenol, thymol and carvacrol performed by Di Pasqua *et al.* [35] revealed alterations in the composition of the fatty acids and the morphology of the cells. The morphology of *E. coli* O157:H7 cells treated with eugenol appeared remarkably altered. This suggests that eugenol may be capable of disrupting the membrane and allowing the leakage of intracellular constituents, while the other compounds may only cause structural alterations of the outer envelope. *S. typhimurium* and *Pseudomonas* spp. cells treated with cinnamaldehyde and limonene presented external modifications, suggesting that these compounds penetrated the cell envelope and altered its structure. Indeed, the *S. typhimurium* cell membrane was altered by carvacrol and thymol, and some of the cells showed swelling after treatment with thymol. *B. thermosphacta* exhibited evident alterations after treatment with cinnamaldehyde, limonene and eugenol; eugenol often induced swelling and occasionally disrupted the external envelope. The substantial fatty acid changes detected by GC analysis was ascribed to a probable alteration of the cell membrane that was observed by SEM. Studies with liposome model systems confirmed that cyclic terpene hydrocarbons accumulated in the membrane, causing a loss of membrane integrity and dissipation of the proton motive force. Observations made by electron microscopy showed that treating *E. coli* O157:H7 with oregano EO, which is rich in thymol and carvacrol, resulted in the collapse of cells after the loss of their contents [4,26,115]. Oussalah*et al.* [92] observed morphological damage and a disruption of the cell membrane after treatment of *E. coli* O157:H7 and *L*. *monocytogenes* with Spanish oregano, Chinese cinnamon and savoury oils. In particular, the *E. coli* cells had holes or white spots on the cell wall. Considerable morphological changes were found on the surface of *P. fluorescens* after treatment with carvacrol and with cinnamaldehyde or with a combination of four different EO vapours containing a high amount of cinnamaldehyde. Negative air ions also resulted in the complete leakage of the cytoplasmic material within a few hours of exposure [116]. Nostro *et al.* [117] and Sandasi *et al.* [118] described the morphological changes of staphylococcal and *Listeria* biofilms after exposure to EOs. The morphological changes of some strains after carvacrol contact were comparable to those described after treatment with other antimicrobial agents, such as antimicrobial peptides [119]. The presence of division septa in treated cells may have been due to the effect of carvacrol on the proteins involved in cell division. This was confirmed by proteomic approaches in *Salmonella* cells treated with thymol [80]. The morphology of the Gram-negative cells was much more affected by carvacrol than the Gram-positive cells due to the presence of an OM in the Gram-negative bacteria and the fact that biological membranes are among the possible targets of carvacrol. However, Gram-negative bacteria are generally considered to be more resistant to EOs. The modified structure of the Gram-negative cell surface could also be interpreted as an adaptive response to stress; in fact, nearly all of the Gram-negative bacteria examined demonstrated greater increase of roughness after carvacrol treatment compared with Gram-positive bacteria. Treatment with carvacrol may increase the exposure of the OM components (e.g., proteins and lipids), causing an increase in roughness. However, in Gram-positive bacteria, carvacrol moves through the peptidoglycan layer and then acts on the cytoplasmic membrane. The structural changes in the membrane, such as fluidity alteration, could lead to a slight modification in the external surface of the Gram-positive cell wall such that they appears less rough but more bumpy than Gram-negative bacteria [120].

### 4.6. Anti-Quorum Sensing Activity

Bacteria coordinate both bacterium-bacterium interactions and associations with higher organisms through intercellular communication systems known as quorum sensing (QS) systems. QS-controlled behaviours occur only when bacteria reach a specific cell density. These behaviours are unproductive if undertaken by a singular bacterium but become effective when the action is simultaneously performed by a group of bacteria. QS can regulate a number of activities, such as virulence factor expression, bioluminescence, sporulation, biofilm formation and mating. The expression of the QS genes results in the production of chemical signalling molecules that are known as autoinducers or bacterial pheromones. These molecules are produced as the bacterial population grows until a threshold concentration perceived by the bacteria is reached, resulting in the activation or repression of specific genes. The accumulation of a stimulatory amount of the QS molecules can occur only when a specific number of cells, referred to as a quorum, are present [121]. Researchers are increasingly investigating herbal products in the quest for new therapeutic and anti-pathogenic agents that might act as nontoxic inhibitors of QS, thus controlling infections without encouraging the appearance of resistant bacterial strains [122]. EOs may represent the richest available reservoir of novel therapeutics [122,123,124]. Bacterial QS may be inhibited through different mechanisms, including (1) the inhibition of AHL synthesis, (2) the inhibition of AHL transport and/or secretion, (3) the sequestration of AHLs, (4) the antagonistic action and (5) the inhibition of targets downstream of AHL receptor binding [125]. Different EOs from ornamental plants have been observed to be effective against biofilms formed by *Salmonella*, *Listeria*, *Pseudomonas*, *Staphylococcus* and *Lactobacillus* spp. Volatile organic compounds, such as those produced by the rhizospheric bacteria *Pseudomonas fluorescens* B-4117 and *Serratia plymuthica* IC1270, may inhibit the cell-cell communication QS network mediated by AHL signalling molecules produced by various bacteria, such as *Agrobacterium*, *Chromobacterium*, *Pectobacterium* and *Pseudomonas*. The EOs of lavender, roses, geraniums, cloves and rosemary are also able to inhibit QS, whereas orange and juniper EOs appear to have no anti-QS properties [126,127,128,129,130,131]. Investigations into the effects of different EO components are in progress. One of the most well-studied EO components is cinnamaldehyde. Its effects have been investigated from diverse points of view, and multiple mechanisms of action have been examined. Niu and Afre [132] observed that the exposure of *V. harveyi* BB886 (the bioluminescence of this strain is induced by 3-hydroxy-C4-HSL) to a concentration of 60 μΜ cinnamaldehyde resulted in a 55% reduction of microbial bioluminescence, and 60% of the bioluminescence of *V. harveyi* BB170 (mediated by AI-2) was reduced at 100 μΜ, again demonstrating that the activity of plant extracts can be strain-specific and may depend on the QS molecule involved. Using the nematode model *Caenorhabditis elegans*, Brackman and colleagues [133,134] demonstrated the ability of 3,4-dichlorocinnamaldehyde to decrease the virulence of *V. anguillarum, V. harveyi* and *V. vulnificus* by affecting the DNA-binding ability of LuxR.

## 5. Conclusions

The action of EOs and their components on bacteria remains a focal area for future research. The study of the synergistic effects among EOs and/or their components could be utilized both to make best use of their antibacterial activity and to reduce their concentrations required to achieve a particular antibacterial effect for food safety and for health purposes.

## References

[1] Abad M.J., Ansuategui M., Bermejo P. (2007). Active antifungal substances from natural sources. ARCHIVOC.

[2] Bakkali F., Averbeck S., Averbeck D., Idaomar M. (2008). Biological effects of essential oils—A review. Food Chem. Toxicol..

[3] Chorianopoulos N.G., Giaouris E.D., Skandamis P.N., Haroutounian S.A., Nychas G.J.E. (2008). Disinfectant test against monoculture and mixed-culture biofilms composed of technological, spoilage and pathogenic bacteria: Bactericidal effect of essential oil and hydrosol of *Satureja thymbra* and comparison with standard acid-base sanitizers. J. Appl. Microbiol..

[4] Burt S.A., Reinders R.D. (2003). Antibacterial activity of selected plant essential oils against *Escherichia coli* O157:H7. Lett. Appl. Microbiol..

[5] De Martino L., de Feo V., Nazzaro F. (2009). Chemical composition and *in vitro* antimicrobial and mutagenic activities of seven lamiaceae essential oils. Molecules.

[6] Trombetta D., Castelli F., Sarpietro M.G., Venuti V., Cristani M., Daniele C., Saija A., Mazzanti G., Bisignano G. (2005). Mechanisms of antibacterial action of three monoterpenes. Antimicrob. Agents Chemother..

[7] Tiwari B.K., Valdramidis V.P., O’Donnel C.P., Muthukumarappan K., Bourke P., Cullen P.J. (2009). Application of natural antimicrobials for food preservation. J. Agric. Food Chem..

[8] Nikaido H. (1994). Prevention of drug access to bacterial targets: Permeability barriers and active efflux. Science.

[9] Vaara M. (1992). Agents that increase the permeability of the outer membrane. Microbiol. Rev..

[10] Plesiat P., Nikaido H. (1992). Outer membranes of Gram-negative bacteria are permeable to steroid probes. Mol. Microbiol..

[11] Nikaido H., Neidhardt F.C. (1996). Outer Membrane. Escherichia coli and Salmonella: Cellular and Molecular biology.

[12] Dorman H.J.D., Deans S.G. (2000). Antimicrobial agents from plants: Antibacterial activity of plant volatile oils. J. Appl. Microbiol..

[13] Helander I.M., Alakomi H.L., Latva K., Mattila-Sandholm T., Pol I., Smid E.J., Gorris L.G.M., von Wright A. (1998). Characterization of the action of selected essential oil components on Gram-negative bacteria. J. Agric. Food Chem..

[14] Helander I.M., Alakomi H.L., Latva-Kala K., Koski P. (1997). Polyethyleneimine is an effective permeabilizer of Gram negative bacteria. Microbiology.

[15] De Martino L., de Feo V., Fratianni F., Nazzaro F. (2009). Chemistry, antioxidant, antibacterial and antifungal activities of volatile oils and their components. Nat. Prod. Comm..

[16] Caballero B., Trugo L.C., Finglas P.M. (2003). Encyclopedia of Food Sciences and Nutrition.

[17] Bagamboula C.F., Uyttendaele M., Debevere J. (2004). Inhibitory effect of thyme and basil essential oils, carvacrol, thymol, estragol, linalool and p-cymene towards *Shigella sonnei* and *S. flexneri.*. Food Microbiol..

[18] Ultee A., Bennik M.H., Moezelaar R. (2002). The phenolic hydroxyl group of carvacrol is essential for action against the food-borne pathogen *Bacillus cereus*. Appl. Environ. Microbiol..

[19] Ben Arfa A., Combes S., Preziosi-Belloy L., Gontard N., Chalier P. (2006). Antimicrobial activity of carvacrol related to its chemical structure. Lett. Appl. Microbiol..

[20] Lambert R.J.W., Skandamis P.N., Coote P.J., Nychas G.J.E. (2001). A study of the minimum inhibitory concentration and mode of action of oregano essential oil, thymol and carvacrol. J. Appl. Microbiol..

[21] Mann C.M., Cox S.D., Markham J.L. (2000). The outer membrane of *Pseudomonas aeruginosa* NCTC 6749 contributes to its tolerance to the essential oil of *Melaleuca alternifolia* (tea tree oil). Lett. Appl. Microbiol..

[22] Aligiannis N., Kalpoutzakis E., Mitaku S., Chinou I.B. (2001). Composition and antimicrobial activity of the essential oils of two *Origanum* species. J. Agric. Food Chem..

[23] Rattanachaikunsopon P., Phumkhachorn P. (2010). Assessment of factors influencing antimicrobial activity of carvacrol and cymene against *Vibrio cholerae* in food. J. Biosci. Bioeng..

[24] Cristani M., D’Arrigo M., Mandalari G., Castelli F., Sarpietro M.G., Micieli D., Venuti V., Bisignano G., Saija A., Trombetta D. (2007). Interaction of four monoterpenes contained in essential oils with model membranes:implications for their antibacterialactivity. J. Agric. Food Chem..

[25] Burt S.A., van der Zee R., Koets A.P., de Graaff A.M., Van Knapen F., Gaastra W., Haagsman H.P., Veldhuizen E.J.A. (2007). Carvacrol induces heat shock protein and inhibits synthesis of flagellin in *Escherichia coli* O157:H7. Appl. Environ. Microbiol..

[26] Sikkema J., de Bont J.A.M., Poolman B. (1995). Mechanisms of membrane toxicity of hydrocarbons. Microbiol. Rev..

[27] Walsh S.E., Maillard J.Y., Russell A.D., Catrenich C.E., Charbonneau D.L., Bartolo R.G. (2003). Activity and mechanisms of action of selected biocidal agents on Gram-positive and-negative bacteria. J. Appl. Microbiol..

[28] Xu J., Zhou F., Ji B.P., Pei R.S., Xu N. (2008). The antibacterial mechanism of carvacrol and thymol against *Escherichia coli*. Lett. Appl. Microbiol..

[29] Turina A.D.V., Nolan M.V., Zygadlo J.A., Perillo M.A. (2006). Natural terpenes: Self-assembly and brane partitioning. Biophys. Chem..

[30] Juven B.J., Kanner J., Schved F., Weisslowicz H. (1994). Factors that Interact with the antibacterial action of thyme essential oil and its active constituents. J. Appl. Bacteriol..

[31] La Storia A., Ercolini D., Marinello F., di Pasqua R., Villani F., Mauriello G. (2011). Atomic force microscopy analysis shows surface structure changes in carvacrol-treated bacterial cells. Res. Microbiol..

[32] Veldhuizen E.J.A., Tjeerdsma-Van Bokhoven J.L.M., Zweijtzer C., Burt S.A., Haagsman H.P. (2006). Structural requirements for the antimicrobial activity of carvacrol. J. Agric. Food Chem..

[33] Ultee A., Kets E.P.W., Alberda M., Hoekstra F.A., Smid E.J. (2000). Adaptation of the food-borne pathogen *Bacillus cereus* to carvacrol. Arch. Microbiol..

[34] Di Pasqua R., Hoskins N., Betts G., Mauriello G. (2006). Changes in membrane fatty acids composition of microbial cells induced by addiction of thymol, carvacrol, limonene, cinnamaldehyde, and eugenol in the growing media. J. Agric. Food Chem..

[35] Di Pasqua R., Betts G., Hoskins N., Edwards M., Ercolini D., Mauriello G. (2007). Membrane toxicity of antimicrobial compounds from essential oils. J. Agric. Food Chem..

[36] Ultee A., Kets E.P.W., Smid E.J. (1999). Mechanisms of action of carvacrol on the food-borne pathogen. Appl. Environ. Microbiol..

[37] Horváth G., Kovács K., Kocsis B., Kustos I. (2009). Effect of thyme (*Thymus vulgaris* L.) essential oil and its main constituents on the outer membrane protein composition of *Erwinia* strains studied with microfluid chip technology. Chromatographia.

[38] Gabel C.V., Berg H.C. (2003). The speed of the flagellar rotary motor of *Escherichia coli* varies linearly with proton motive force. Proc. Natl. Acad. Sci. USA.

[39] Laekeman G.M., VanHoof L., Haemers A., Berghe D.A.V., Herman A.G., Vlietinck A.J. (1990). Eugenol a valuable compound for *in vitro* experimental research and worthwhile for further *in vivo* investigation. Phytother. Res..

[40] Jung H.G., Fahey G.C. (1983). Nutritional implications of phenolic monomers and lignin: A review. J. Anim. Sci..

[41] Pauli A., Kubeczka K.H. (2010). Antimicrobial properties of volatile phenylpropanes. Nat. Prod. Commun..

[42] Zemek J., Kosikova B., Augustin J., Joniak D. (1979). Antibiotic properties of lignin components. Folia Microbiol..

[43] Zemek J., Valent M., Pódová M., Košíková B., Joniak D. (1987). Antimicrobial properties of aromatic compounds of plant origin. Folia Microbiol..

[44] Hyldgaard M., Mygind T., Rikke L.M. (2012). Essential oils in food preservation: Mode of action, synergies, and interactions with food matrix components. Front. Microbiol..

[45] Gill A.O., Holley R.A. (2004). Mechanisms of bactericidal action of cinnamaldehyde against *Listeria monocytogenes* and of eugenol against *L. monocytogenes* and *Lactobacillus sakei*. Appl. Environ. Microbiol..

[46] Thoroski J. (1989). Eugenol induced inhibition of extracellular enzyme production by *Bacillus cereus*. J. Food Prot..

[47] Wendakoon C.N., Sakaguchi M. (1995). Inhibition of amino acid decarboxylase activity of *Enterobacter aerogenes* by active components in spices. J. Food Prot..

[48] Fitzgerald D.J., Stratford M., Gasson M.J., Ueckert J., Bos A., Narbad A. (2004). Mode of antimicrobial of vanillin against *Escherichia coli*, *Lactobacillus plantarum* and *Listeria innocua*. J. Appl. Microbiol..

[49] Fitzgerald D.J., Stratford M., Gasson M.J., Narbad A. (2005). Structure-function analysis of the vanillin molecule and its antifungal properties. J. Agric. Food Chem..

[50] Oosterhaven K., Poolman B., Smid E.J. (1995). S-carvone as a natural potato sprout inhibiting, fungistatic and bacteristatic compound. Ind. Crops Prod..

[51] Rauha J.P., Remes S., Heinonen M., Hopia A., Khkönen M., Kujala T., Pihlaja K., Vuorela H., Vuorela P. (2000). Antimicrobial effects of Finnish plant extracts containing flavonoids and other phenolic compounds. Int. J. Food Microbiol..

[52] Holley R.A., Patel D. (2005). Improvement in shelf-life and safety of perishable foods by plant essential oils and smoke antimicrobials. Food Microbiol..

[53] Lis-Balchin M., Deans S.G., Eaglesham E. (1998). Relationship between bioactivity and chemical composition of commercial essential oils. Flavour Fragr. J..

[54] Davidson P.M., Beuchat M.P., Montville L.R. (2001). Chemical Preservatives and Naturally Antimicrobial Compounds. Food Microbiology. Fundamentals and Frontiers.

[55] Pina-Vaz C., Gonçalves Rodrigues A., Pinto E., Costa-de-Oliveira S., Tavares C., Salgueiro L., Cavaleiro C., Gonçalves M.J., Martinez-de-Oliveira J. (2004). Antifungal activity of *Thymus* oils and their major compounds. J. Eur. Acad. Dermatol. Venereol..

[56] Gutierrez J., Barry-Ryan C., Bourke P. (2008). The anti-microbial efficacy of plant essential oil combinations and interactions with food ingredients. Int. J. Food Microbiol..

[57] Burt S. (2004). Essential oils: their antibacterial properties and potential applications in foods—A review. Int. J. Food Microbiol..

[58] Marino M., Bersani C., Comi G. (2001). Antimicrobial activity of the essential oils of *Thymus vulgaris* L. measured using a bioimpedimetric method. Int. J. Food Microbiol..

[59] Poolman B., Driessen A.J.M., Konings W.N. (1987). Regulation of solute transport in Streptococci by external and internal pH values. Microbiol. Rev..

[60] Trumpower B.L., Gennis R.B. (1994). Energy transduction by cytochrome complexes in mitochondrial and bacterial respiration: the enzymology of coupling electron transfer reactions to transmembrane proton translocation. Ann. Rev. Biochem..

[61] Andrews R.E., Parks L.W., Spence K.D. (1980). Some effects of Douglas fir terpenes on certain microorganisms. Appl. Environ. Microbiol..

[62] Uribe S., Ramirez T., Pena A. (1985). Effects of β-pinene on yeast membrane functions. J. Bacteriol..

[63] Knobloch K., Pauli A., Iberl B. (1988). Antibacterial activity and antifungal properties of essential oil components. J. Essent. Oils Res..

[64] Gill A.O., Holley R.A. (2006). Disruption of *E. coli*, *Listeria monocytogenes* and *Lactobacillus sakei* cellular membranes by plant oil aromatics. Int. J. Food Microbiol..

[65] Gustafson J.E., Liew Y.C., Chew S., Markham J.L., Bell H.C., Wyllie S.G., Warmington J.R. (1998). Effects of tea tree oil on *Escherichia coli*. Lett. Appl. Microbiol..

[66] Ultee A., Smid E.J. (2001). Influence of carvacrol on growth and toxin production by Bacillus cereus. Int. J. Food Microbiol..

[67] Kim J., Marshal M.R., Wie C.I. (1995). Antibacterial activity of some essential oils components against five foodborne pathogens. J. Agric. Food Chem..

[68] Tassou C., Koutsoumanis K., Nychas J.E. (2000). Inhibition of *Salmonella enteritidis* and *Staphylococcus aureus* in nutrient broth by mint essential oil. Food Res. Int..

[69] Mrozik A., Pietrovska-Seget Z., Labuzek S. (2004). Changes in whole cell-derived fatty acids induced by naphthalene in bacteria from genus *Pseudomonas*. Microbiol. Res..

[70] Carson C.F., Mee B.J., Riley T.V. (2002). Mechanism of action of *Melaleuca alternifolia* (tea tree) oil on *Staphylococcus aureus* determined by time-kill, lysis, leakage, and salt tolerance assays and electron microscopy. Antimicrob. Agents Chemother..

[71] Heath R.J., Rock C.O. (2004). Fatty acid biosynthesis as a target for novel antibacterials. Curr. Opin. Invest. Drugs.

[72] Heath R.J., White S.W., Rock C.O. (2001). Lipid biosynthesis as a target for antibacterial agents. Prog. Lipid Res..

[73] Campbell J.W., Cronan J.E. (2001). Bacterial fatty acids biosynthesis: Targets for antibacterial drug discovery. Ann. Rev. Microbiol..

[74] Bayer A.S., Presad R., Chandra J., Smirti A.M., Varma A., Skurray R.A., Firth N., Brown M.H., Koo S.P., Yeaman M.R. (2000). *In vitro* resistance of *Staphylococcus aureus* to thrombin induced platelet microbicidal protein is associated with alterations in cytoplasmic membrane fluidity. Infect. Immun..

[75] Heath R.J., Jackowski S., Rock C.O., Vance J.E., Vance D.E. (2002). Fatty Acid and Phospholipid Metabolism in Prokaryotes. Biochemistry of Lipids, Lipoproteins and Membranes.

[76] Russell N.J. (1984). Mechanism of thermal adaptation in bacteria: Blueprints for survival. Tr. Biochem. Sci..

[77] Russell N.J. (1997). Psychrophilic bacteria: Molecular adaptations of membrane lipids. Comp. Biochem. Physiol..

[78] Heipieper H.J., Meinhardt F., Segura A. (2003). The cis-trans isomerase of unsaturated fatty acids in *Pseudomonas* and *Vibrio*: Biochemistry, molecular biology and physiological function of a unique stress adaptive mechanism. FEMS Microbiol. Lett..

[79] Domadia P., Swarup S., Bhunia A., Sivaraman J., Dasgupta D. (2007). Inhibition of bacterial cell division protein FtsZ by cinnamaldehyde. Biochem. Pharmacol..

[80] Di Pasqua R., Mamone G., Ferranti P., Ercolini D., Mauriello G. (2010). Changes in the proteome of *Salmonella enterica* serovar Thompson as stress adaptation to sublethal concentrations of thymol. Proteomics.

[81] Kumar M., Berwal J.S. (1998). Sensitivity of food pathogens to garlic (*Allium sativum*). J. Appl. Microbiol..

[82] Di Pasqua R., Mauriello G., Mamone G., Ercolini D. (2013). Expression of DnaK, HtpG, GroEL and Tf chaperones and the corresponding encoding genes during growth of *Salmonell*a Thompson in presence of thymol alone or in combination with salt and cold stress. Food Res. Int..

[83] Baucheron S., Mouline C., Praud K., Chlaus-Dancla E., Cloeckaert A. (2005). TolC but not AcrB is essential for multidrugresistant *Salmonella enterica* serotype *Typhimurium* colonization of chicks. J. Antimicrob. Chem..

[84] Klose K.E., Mekalanos J.J. (1997). Simultaneous prevention of glutamine synthesis and high-affinity transport attenuates *Salmonella typhimurium* virulence. Infect. Immun..

[85] Miesel L., Greene J., Black T.A. (2003). Genetic strategies for antibacterial drug discovery. Nat. Rev. Gen..

[86] Xu H.H., Trawick J.D., Haselbeck R.J., Forsyth R., Yamamoto R.T., Archer R., Patterson J., Allen M., Froelich1 J.M. , Taylor I. (2010). *Staphylococcus aureus* Target Array: Comprehensive differential essential gene expression as a mechanistic tool to profile antibacterials. Antimicrob. Agents Chemother..

[87] Scotti P.A., Urbanus M.L., Brunner J., de Gier J.W.L., von Heijne G., van der Does C., Driessen A.J.M., Oudega B., Luirink J. (2000). YidC, the *Escherichia coli* homologue of mitochondrial Oxa1p, is a component of the Sec translocase. EMBO J..

[88] Serek J., Bauer-Manz G., Struhalla G., van den Berg L., Kiefer D., Dalbey R., Kuhn A. (2004). *Escherichia coli* YidC is a membrane insertase for Sec-independent proteins. EMBO J..

[89] Samuelson J.C., Chen M., Jiang F., Möller I., Wiedmann M., Kuhn A., Phillips G.J., Dalbey R.E. (2000). YidC mediates membrane protein insertion in bacteria. Nature.

[90] Van der Laan M., Urbanus M., Ten Hagen-Jongman C., Nouwen N., Oudega B., Harms N., Driessen A.J.M., Luirink J. (2003). A conserved function of YidC in the biogenesis of respiratory chain complexes. Proc. Nat. Acad. Sci. USA.

[91] Patil S.D., Sharma R., Srivastava S., Navani N.K., Pathania R. (2013). Down regulation of yidC in *Escherichia coli* by antisense RNA expression results in sensitization to antibacterial essential oils eugenol and carvacrol. PLoS One.

[92] Oussalah M., Caillet S., Saucier L., Lacroix M. (2006). Antimicrobial effects of selected plant essential oils on the growth of a *Pseudomonas putida* strain isolated from meat. Meat Sci..

[93] Turgis M., Han J., Caillet S., Lacroix M. (2009). Antimicrobial activity of mustard essential oil against *Escherichia coli* O157:H7 and *Salmonella typhi*. Food Control..

[94] Caillet S., Ursachi L., Shareck F., Lacroix M. (2009). Effect of gamma radiation and oregano essential oil on murein and ATP concentration of *Staphylococcus aureus*. J. Food Sci..

[95] Caillet S., Lacroix M. (2006). Effect of gamma radiation and oregano essential oil on murein and ATP concentration of *Listeria monocytogenes*. J. Food Prot..

[96] Abee T., Klaenhammer T.R., Letellier L. (1994). Kinetic studies of the action of lactacin F, a bacteriocin produced by *Lactobacillus johnsonii* that forms poration complexes in the cytoplasmic membrane. Appl. Env. Microbiol..

[97] Shabala L., Budde L., Ross B., Siegumfeldt T., Jakobsen H., McMeekin M. (2002). Responses of *Listeria monocytogenes* to acid stress and glucose availability revealed by a novel combination of fluorescence microscopy and microelectrode ion-selective techniques. Appl. Env. Microbiol..

[98] Kwon J.A., Yu C.B., Park H.D. (2003). Bactericidal effects and inhibition of cell separation of cinnamic aldehyde on *Bacillus cereus*. Lett. Appl. Microbiol..

[99] Carneiro S., Villas-Bôas S.G., Ferreira E.C., Rocha I. (2011). Metabolic footprint analysis of recombinant *Escherichia coli* strains during fed-batch fermentations. Mol. Bio. Syst..

[100] Van der Werf M.J., Overkamp K.M., Muilwijk B., Coulier L., Hankemeier T. (2007). Microbial metabolomics: Toward a platform with full metabolome coverage. Anal. Biochem..

[101] Rabinowitz J.D. (2007). Cellular metabolomics of *Escherichia coli*. Exp. Rev. Prot..

[102] Mapelli V., Olsson L., Nielsen J. (2008). Metabolic footprinting in microbiology: Methods and applications in functional genomics and biotechnology. Trends Biotech..

[103] Jozefczuk S., Klie S., Catchpole G., Szymanski J., Cuadros-Inostroza A., Steinhauser D., Selbig J., Willmitzer L. (2010). Metabolomic and transcriptomic stress response of *Escherichia coli*. Mol. Syst. Biol..

[104] Gunasekera T.S., Csonka L.N., Paliy O. (2008). Genome-wide transcriptional responses of *Escherichia coli* K-12 to continuous osmotic and heat stresses. J. Bacteriol..

[105] Durfee T., Hansen A.M., Zhi H., Blattner F.R., Jin D.J. (2008). Transcription profiling of the stringent response in *Escherichia coli*. J. Bacteriol..

[106] Malin G., Lapidot A. (1996). Induction of synthesis of tetrahydropyrimidine derivatives in *Streptomyces* strains and their effect on *Escherichia coli* in response to osmotic and heat stress. J. Bacteriol..

[107] Picone G., Laghi L., Gardini F., Lanciotti R., Siroli L., Capozzi F. (2013). Evaluation of the effect of carvacrol on the *Escherichia coli* 555 metabolome by using 1H-NMR spectroscopy. Food Chem..

[108] Cox S.D., Gustafson J.E., Mann C.M., Markham J.L., Liew Y.C., Hartland R.P. (1998). Tea tree oil causes K+ leakage and inhibits respiration in *Escherichia coli*. Lett. Appl. Microbiol..

[109] Hossain Z.S.M., Bojko B., Pawliszyn J. (2013). Automated SPME–GC–MS monitoring of headspace metabolomic responses of *E. coli* to biologically active components extracted by the coating. An. Chim. Acta.

[110] Hafedh H., Najla T., Emira N., Mejdi S., Hanen F., Riadh K., Amina B. (2009). Biological activities of the essential oils and methanol extract of two cultivated mint species (*Mentha longifolia* and *Mentha pulegium*) used in the Tunisian folkloric medicine. World J. Biotec. Microbiol..

[111] Kalchayanand N., Dunneb P., Sikes A., Ray B. (2004). Viability loss and morphology change of foodborne pathogens following exposure to hydrostatic pressures in the presence and absence of bacteriocins. Int. J. Food Microbiol..

[112] Braga P.C., Ricci D. (1998). Atomic Force Microscopy: Application to investigation of *Escherichia coli* morphology before and after exposure to cefodizime. Antimicrob. Agents Chemother..

[113] Slavik M.F., Kim W.J., Walker J.T. (1995). Reduction of Salmonella and Campylobacter on chicken carcasses by changing scalding temperature. J. Food Prot..

[114] Sikkema J., Weber F.J., Heipieper H.J., de Bont J.A.M. (1994). Cellular toxicity of lipophilic compounds: Mechanisms, implications, and adaptations. Biocatalysis.

[115] De Sousa J.P., de Araújo Torres R., Alves de Azerêdo G., Queiroz Figueiredo B.R.C., da Silva Vasconcelos M.A., Leite de Souza E. (2012). Carvacrol and 1,8-cineole alone or in combination at sublethal concentrations induce changes in the cell morphology and membrane permeability of *Pseudomonas fluorescens* in a vegetable-based broth. Int. J. Food Microbiol..

[116] Tyagi A.K., Malik A. (2010). Antimicrobial action of essential oil vapours and negative air ions against *Pseudomonas fluorescens*. Int. J. Food Microbiol..

[117] Nostro A., Marino A., Blanco A.R., Cellini L., di Giulio M., Pizzimenti F., Roccaro A.S., Bisignano G. (2009). *In vitro* activity of carvacrol against staphylococcal preformed biofilm by liquid and vapour contact. J. Med. Microbiol..

[118] Sandasi M., Leonard C.M., Viljoen A.M. (2008). The effect of five common essential oil components on *Listeria monocytogenes* biofilms. Food Control.

[119] Meincken M., Holroyd D.L., Rautenbach M. (2005). Atomic force microscopy study of the effect of antimicrobial peptides on the cell envelope of *Escherichia coli*. Antimicrob. Agents Chemother..

[120] Alakomi H.L., Paananen A., Suihko M.L., Helander I.M., Saarela M. (2006). Weakening effect of cell permeabilizer on gram-negative bacteria causing biodeterioration. Appl. Environ. Microbiol..

[121] Bassler B.L. (2002). Small talk: Cell-to-cell communication in bacteria. Cell.

[122] Hentzer M., Givskov M. (2003). Pharmacological inhibition of quorum sensing for the treatment of chronic bacterial infections. J. Clin. Invest..

[123] Lewis K., Ausubel F.M. (2006). Prospects of plant derived antibacterials. Nat. Biotechnol..

[124] Kumar V.P., Chauhan N.S., Rajani H.P.M. (2006). Search for antibacterial and antifungal agents from selected Indian medicinal plants. J. Ethnopharmacol..

[125] Nazzaro F., Fratianni F., Coppola R. (2013). Quorum sensing and phytochemicals. Int. J. Mol. Sci..

[126] Faleiro M.L., Méndez-Vilas A. (2011). The Mode of Antibacterial Action of ESsential Oils. Science Against Microbial Pathogens: Communicating Current Research and Technological Advances.

[127] Al-Shuneigat J., Cox S.D., Markham J.L. (2005). Effects of a topical essential oil-containing formulation on bio-film-forming coagulase-negative staphylococci. Lett. Appl. Microbiol..

[128] Khan M.S., Zahin M., Hasan S., Husain F.M., Ahmad I. (2009). Inhibition of quorum sensing regulated bacterial functions by plant essential oils with special reference to clove oil. Lett. Appl. Microbiol..

[129] Zaki A.A., Shaaban M.I., Hashish N.E., Amer M.A., Lahloub M.F. (2013). Assessment of anti-quorum sensing activity for some ornamental and medicinal plants native to Egypt. Sci. Pharm..

[130] Szabó M.A., Varga G.Z., Hohmann J., Schelz Z., Szegedi E., Amaral L., Molnár J. (2010). Inhibition of quorum-sensing signals by essential oils. Phytother. Res..

[131] Chernin L.S., Winson M.K., Thompson J.M., Haran S., Bycroft B.W., Chet I., Williams P., Gordon S., Stewart A.B. (1998). Chitinolytic activity in *Chromobacterium violaceum*: Substrate analysis and regulation by quorum sensing. J. Bacteriol..

[132] Niu S., Afre S., Gilbert E.S. (2006). Subinhibitory concentrations of cinnamaldehyde interfere with quorum sensing. Lett. Appl. Microbiol..

[133] Brackman G., Celen S., Hillaert U., Calenbergh S.V., Cos P., Maes L., Nelis H.J., Coenye T. (2011). Structure-activity relationship of cinnamaldehyde analogs as inhibitors of ai-2 based quorum sensing and their effect on virulence of *Vibrio* spp.. PLoS One.

[134] Brackman G., Defoirdt T., Miyamoto C., Bossier P., Calenbergh S.V., Nelis H., Coenye T. (2008). Cinnamaldehyde and cinnamaldehyde derivatives reduce virulence in *Vibrio* spp. by decreasing the DNA-binding activity of the quorum sensing response regulator LuxR. BMC Microbiol..

